# Data from a UK-based multicentre randomised feasibility study investigating the management of post-traumatic benign paroxysmal positional vertigo: mixed-methods analyses

**DOI:** 10.1136/bmjopen-2026-117657

**Published:** 2026-06-16

**Authors:** Rebecca M Smith, Bithi Sahu, Caroline Burgess, Jenna Beattie, Abby Newdick, Vassilios Tahtis, Jeremy Corcoran, Jonathan Marsden, Barry M Seemungal

**Affiliations:** 1 Centre of Vestibular Neurology, Imperial College London, London, UK; 2 Population Health Sciences, King’s College London, London, UK; 3 Imperial College Healthcare NHS Foundation Trust, London, UK; 4 St. George’s Hospital NHS Foundation Trust, London, UK; 5 Kings’ College Hospital NHS FoundationTrust, London, UK; 6 Guy's and St Thomas’ NHS Foundation Trust, London, UK; 7 School of Health Professions, University of Plymouth, Plymouth, UK

**Keywords:** Feasibility Studies, Rehabilitation medicine, Neurotology, Brain Injuries

## Abstract

**Objectives:**

Traumatic brain injury commonly causes dizziness and balance problems. Benign paroxysmal positional vertigo (BPPV) is the most frequent cause of inner-ear related post-traumatic vestibular dysfunction. However, optimal assessment and treatment practices are poorly evidenced. Using a mixed methods approach, we aimed to explore the feasibility of managing post-traumatic BPPV.

**Design:**

A mixed-methods randomised feasibility study. In this paper, quantitative and qualitative data relating to key feasibility targets around recruitment, randomisation and assessment and treatment were selected and integrated to provide an in-depth understanding of BPPV management.

**Setting:**

Three UK major trauma centres

**Participants:**

Hospitalised adults with post-traumatic BPPV.

**Interventions:**

Patients were randomised to one of three interventions (repositioning manoeuvres, Brandt-Daroff exercises and advice).

**Results:**

Screening and recruitment varied between sites due to individual and site-specific factors. Randomisation to advice was experienced differently by healthcare professionals and patients, suggesting that changes to the study design of a future effectiveness trial may be required. Assessment and treatment were acceptable to participants, supporting advancement to an effectiveness trial.

**Conclusions:**

Integration of qualitative and quantitative data revealed new findings relating to the primary outcomes of the feasibility study, while strengths and weaknesses of the trial design were also elucidated. Taken together, such data will influence and enhance the design of a future trial.

**Trial registration number:**

ISRCTN91943864.

**STRENGTHS AND LIMITATIONS OF THE STUDY**

- This paper uses a mixed-methods approach to report quantitative and qualitative trial data from a multicentre feasibility study investigating assessment and treatment of benign paroxysmal positional vertigo in acute traumatic brain injury.
- Through integrating both qualitative and quantitative trial data pertaining to key feasibility outcomes, key strengths and weaknesses of the trial design have been elucidated.
- Robust integration methods common in mixed-methods trials, such as joint displays, are used to provide a visual representation of the results.

## Background

 Traumatic brain injury (TBI) is the the most common cause of morbidity and mortality in adults under 40 years in the UK.^1^ Post-traumatic vestibular symptoms such as dizziness or imbalance are frequent and are caused by peripheral (inner ear) and/or central (brain) vestibular dysfunction.^2 3^ Long-lasting vestibular symptoms are linked to reduced health-related quality of life, physical and psychological impairments,^4^ as well as increased risk of falls.^5^ Falls have been linked to excess mortality in community dwelling TBI survivors^6^ and are associated with high healthcare costs,^7^ justifying the rationale for treating modifiable risk factors for falls.

The most frequent cause of *peripheral* vestibular dysfunction following acute traumatic brain injury is benign paroxysmal positional vertigo (BPPV).^2 8 9^ BPPV is a condition in which small ‘crystals’ or otoconia are displaced into one or more of three semi-circular canals. During a provocative test, a Dix-Hallpike or Supine head roll test, movement of the otoconia causes a characteristic eye movement or nystagmus,^10^ and sometimes, but not always, an associated sensation of vertigo.^9 11^ Despite it being so common, post-traumatic BPPV is not typically managed by ward healthcare professionals acutely.^12^ Delays are problematic, with a noteworthy impact on quality of life,^13^ an association with falls^14^ and a higher burden of symptoms.^15^ There is evidence in idiopathic BPPV to support early treatment;^10 16 17^ however, this is yet to be translated to the TBI population. Repositioning manoeuvres are recommended for treatment of idiopathic BPPV;^10^ however, Brandt-Daroff exercises and watchful waiting, with patient education, continue to be used as treatment options,^18^ despite unfavourable evidence.^10^

Qualitative research has shown barriers to managing BPPV in this population include role and knowledge-based factors, as well as healthcare professionals’ concerns regarding the practicalities of assessment and treatment procedures.^12^ Perhaps due to these factors, there are no prior acute, prospective trials investigating the most effective intervention for acute post-traumatic BPPV. Accordingly, a mixed-methods randomised feasibility study was conducted to investigate the safety, practicability and tolerability of assessing and treating acute post-traumatic BPPV.^19^ The quantitative results and qualitative findings have been published separately.^8 20^

**Figure 1:**
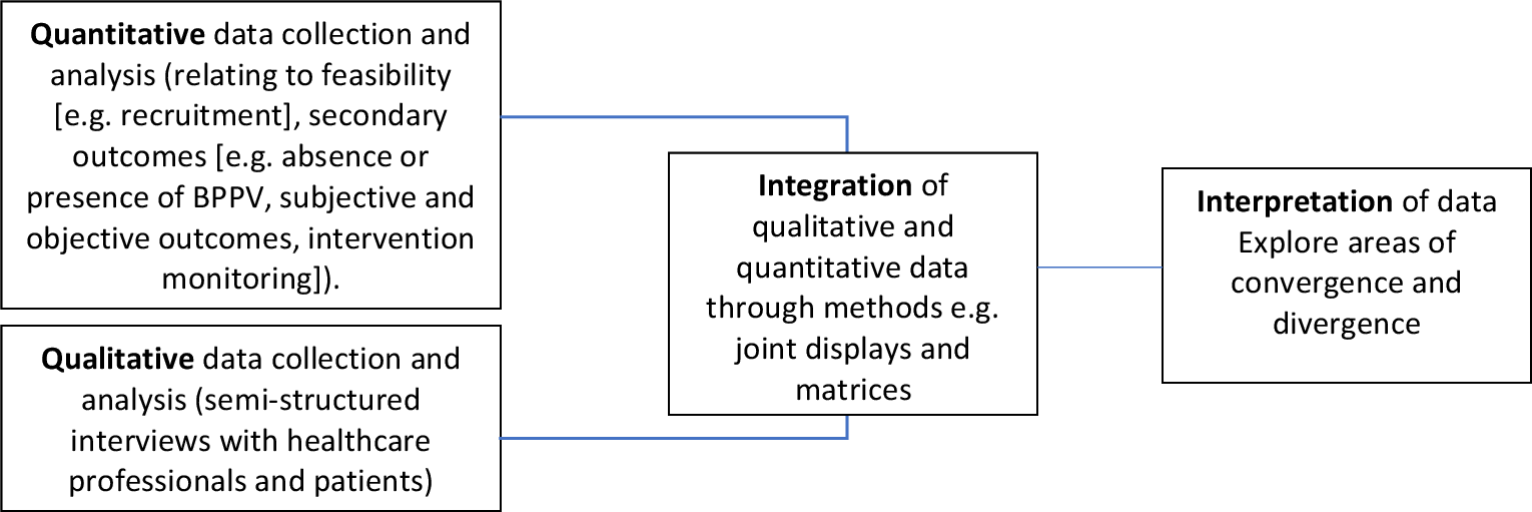
Figure showing how qualitative and quantitative data were collected and integrated in a convergent design. BPPV, benign paroxysmal positional vertigo.

The present study aimed to combine quantitative and qualitative data from the trial (figure 1) to increase depth and breadth of understanding and facilitate a more comprehensive approach to determining trial feasibility^21^ and progression,^22^ key processes in designing complex interventions.^23^ Advantages of integrating the two forms of data include the reduction of risk of conveying misleading conclusions^24^ and ‘development or facilitation’ whereby analysis of one data set enhances interpretation of the other.^25^ To rationalise which findings were appropriate to integrate, trial progression criteria (proportions of patients eligible, consenting and withdrawing) and key objectives of the feasibility study (establish trial recruitment and retention rates, explore the acceptability of randomisation, explore the fidelity and acceptability of the interventions and investigate missing data and adverse events (AEs) relating to treatment) were revisited. Based on these objectives, areas chosen for integration were recruitment, randomisation and assessment and treatment.

## Methods

### Design

This was a multicentre randomised three-armed parallel groups mixed-methods feasibility trial. Acute TBI patients, who tested positive for BPPV, were sequentially randomised to one of three interventions (repositioning manoeuvres (therapist-led movements of the head and body), Brandt-Daroff exercises (patient-led movements of the head and body) and advice (verbal and written advice relating to sitting or standing up slowly and being adequately hydrated)). All interventions were delivered during the hospital stay by trained therapists. Repositioning manoeuvres included an Epley or Semont manoeuvre for posterior canal BPPV or supine log-roll for horizontal canal BPPV.^19^ Patients in the Brandt-Daroff group received two therapist-supervised sessions and were instructed to continue with the exercises two times per day for a total of 2 weeks. Those in the advice group received two therapist-led sessions comprising written and verbal advice. Therapists were instructed to reassess all patients for BPPV prior to discharge. Further trial design and methodological details can be found in prior publications.^8 19^

### Patient and public involvement

The trial was designed and delivered following discussions with patients, who reported that acute care for patients with post-traumatic vestibular dysfunction was not optimal. During the trial, a steering group which included two patient and public members made key decisions about trial delivery and dissemination.

### Setting and participants

Participants were patients and healthcare professionals recruited from three London major trauma centres. Patient participants were adults over the age of 18 years, in-patient on a major trauma or outlying rehabilitation ward and with a closed traumatic brain injury.^26^ Patients were excluded if they were medically unstable, had a current or previous history of substance or alcohol abuse or had cervical orthopaedic or vascular instability precluding BPPV assessment. Healthcare professional participants were physiotherapists, occupational therapists or research nurses. Therapists (inclusive of physiotherapists and occupational therapists) had trial roles involving screening, consenting, outcome measure completion, randomisation or assessment and treatment.^19^ Research nurses’ trial roles included screening, consenting and outcome measure completion.

### Data collection—quantitative

Consenting patients, regardless of dizziness complaints, were screened for BPPV. Those testing positive completed questionnaires pertaining to burden of dizziness and imbalance (Dizziness Handicap Inventory (DHI),^27^ University California Los Angeles Dizziness Questionnaire (UCLA-DQ),^28^ Activities specific Balance Confidence scale (ABC)^29^), general health (EQ-5D^30^), TBI recovery (Glasgow Outcome Score Extended^31^) and mood (Hospital Anxiety and Depression Scale^32^), and were asked to complete bedside tests of static (modified clinical test of sensory interaction in balance^33^) and dynamic balance (Modified Dynamic Gait Index^34^). Following randomisation and treatment, patients were followed up at 4 and 12 weeks with repeat BPPV testing (by a blinded examiner), subjective questionnaires and objective balance tests. AEs of falls were captured by patients in diaries, while AEs of vomiting were captured by therapists.

### Data collection—qualitative

All patients, regardless of site or treatment allocation, were eligible to take part in a semi-structured interview at the end of the trial. Interviews were audio-recorded and were conducted either via face-to-face or via video for pragmatic reasons. Purposive sampling was used to gather a cohort of patients who had been recruited across all three sites and all three treatment arms. Individual interviews were conducted with the chief investigator (RMS) and utilised a topic guide (online supplemental material 1).

All healthcare professionals with a role in the study were eligible to take part. Purposive sampling was used to gather a sample of healthcare professionals with a range of trial roles, across all treatment sites and all professional groups. Healthcare professional interviews were conducted by the same interviewer (BS) and used a different topic guide (online supplemental material 2). The theoretical domains framework (TDF) was utilised to inform separate topic guides for patient and healthcare professional interviews. In this study, the TDF was used to explore barriers or facilitators to trial participation, implementation of the interventions and potential changes to trial delivery.^35 36^ Investigators completing interviews (RMS and BS) had prior training in conducting qualitative interviews.

### Data analysis—quantitative data

Normality of data was assessed using graphical and statistical methods. For normally distributed data, the means and SD are reported. For non-normally distributed data, medians and IQRs are reported. ORs were used to evaluate categorical frequency. Mann Whitney U test (non-normally distributed data) and two-way mixed ANOVA, where Group was the between-subjects factor (Manoeuvres vs Brandt-Daroff vs Advice) and Time was the within-subjects factor (Baseline vs Follow-up), analysed treatment effects. Analyses were performed using R Statistical Software (V.2022.12.0; R Core Team 2021).

### Data analysis—qualitative data

Data were analysed inductively using the Framework approach comprising a series of five steps whereby codes are applied to each transcript and reduced into groups or themes.^37^ Separate frameworks were utilised for healthcare professional and patient data due to the different nature of the populations. To heighten rigour and transparency of results, two researchers (RMS and BS) coded the transcripts. Framework drafts were discussed among the research team and refined until a consensus regarding the Framework was reached.

### Data integration

Figure 1 details how qualitative and quantitative data were collected and then integrated. Several approaches may be used to integrate qualitative and quantitative data sets including joint displays, data transformation and mixed-methods matrices.^38 39^ Despite the variety of approaches, there appears to be a common aim: to consider and identify where data converge, contain contradictions, or are complementary. Joint displays or mixed-methods matrices have been commonly used in trials research^39 40^ due to their ability to (1) compare and contrast data from individuals, groups or organisations and (2) develop new insights or inferences.^41^ Therefore, in this study, methods for data integration included the use of joint displays and matrices, noting recommendations from Guetterman et al. ^42^ such as labelling quantitative and qualitative data, being consistent with design and integration approaches, and identifying inferences from the display.

## Results

### Recruitment and clinical characteristics of trial patients

Of 2014 patients screened for eligibility, 196 gave consent and 180 were assessed for BPPV (see online supplemental material 3 for CONsolidated Standards Of Reporting Trial (CONSORT) flow diagram). Consent rates varied between sites (29%–52%) and are shown in depth in online supplemental material 4); 62/180 (34%) were diagnosed with BPPV; however, four patients were discharged prior to randomisation and treatment, thus 58 were enrolled. Clinical characteristics of the 58 participants are noted in table 1. The mean age of the sample was 53 years with a male predominance (67%). Seventy-eight per cent had a moderate-severe TBI. Thirty patients (52%) had a unilateral posterior canal BPPV, while 23/58 (40%) had bilateral posterior canal BPPV. A smaller proportion (9%) had mixed horizontal and posterior canal BPPV. Length of stay was not statistically different between groups (p*=*0.06). Four patients (4/58; 7%) dropped out or withdrew from the study (2/20; 10% from the manoeuvre group and 2/19; 11% from the advice group). Demographic details of patients and healthcare professionals participating in trial interviews are noted in tables2  3.

**Table 1:** Clinical characteristics of patients taking part in the feasibility study (n=58)

Clinical characteristic	Manoeuvres (20)	Brandt-Daroff (19)	Advice (19)
Age in years, mean (SD)	53 (15)	53 (18)	55 (20)
Gender, n (%)			
Male	14 (70%)	15 (79%)	10 (53%)
Female	6 (30%)	4 (21%)	9 (47%)
Injury details			
GCS, median (IQR)	14 (1.5)	14 (2.5)	15 (1)
Mechanism of injury–fall, n (%)	8 (40%)	9 (47%)	8 (42%)
Skull fractures, n (%)	13 (65%)	13 (68%)	10 (53%)
Moderate–severe TBI, n (%)	14 (70%)	11 (57%)	15 (79%)
BPPV typology			
Unilateral BPPV, n (%)	8 (40%)	13 (68%)	9 (47%)
Bilateral BPPV, n (%)	9 (45%)	4 (21%)	10 (53%)
Mixed BPPV, n (%)	3 (15%)	2 (11%)	0 (0%)
Baseline clinical variables			
DHI, median (IQR)	36 (26–72)	15 (7.5–44.5)	22 (16–56)
FAC, independent, n (%)	10 (50%)	12 (63%)	12 (63%)
BPPV resolution at 12 weeks (%)	14/18 (78%)	8/19 (42%)	9/17 (53%)
Length of stay—days, median (IQR)	13.5 (6–23)	7 (3–17)	11 (5–19)

BPPV, benign paroxysmal positional vertigo; DHI, Dizziness Handicap Inventory; FAC, functional ambulation category (six point clinician rated scale ranging from non-functional to independent on all surfaces); Mixed BPPV, concurrent posterior and horizontal canal benign paroxysmal positional vertigo; GCS (at scene), Glasgow Coma Score.

**Table 2:** Demographics of 15 healthcare professionals participating in semistructured interviews

Healthcare professional (n) site	Number interviewed	Mean age in years (SD)	Sex (females/males)
Physiotherapist (7)			
Site A	3	33 (4)	2/1
Site B	3	30 (4)	3/0
Site C	1	27	1/0
Occupational therapist (4)			
Site A	1	37	1/0
Site B	0	N/A	0
Site C	3	39 (5)	2/1
Research nurse (4)			
Site A	4	33 (5)	3/1

N/A, not applicable.

**Table 3:** Demographics of 26 patients participating in semistructured interviews

Intervention (n)	Interviewed (n)	Male (n,%)	Mean age in years (SD)	Resolved BPPV (n,%)
Manoeuvres (9)				
Site A	5	3 (60%)	53 (17)	4 (80%)
Site B	2	1 (50%)	35 (18)	0
Site C	2	2 (100%)	58 (37)	2 (100%)
Brandt-Daroff (8)				
Site A	4	2 (50%)	68 (9)	1 (25%)
Site B	3	2 (66%)	57 (5)	2 (66%)
Site C	1	1 (100%)	55	0
Advice (9)				
Site A	2	2 (100%)	74 (10)	2 (100%)
Site B	6	6 (100%)	58 (18)	4 (66%)
Site C	1	1 (100%)	48	0

BPPV, benign paroxysmal positional vertigo.

### Summary of quantitative and qualitative findings

#### Quantitative findings

Resolution of BPPV at 12 weeks occurred in 35/58 (60%). AEs of falls (table 4) and vomiting (table 5) were equally distributed throughout intervention arms. Intervention efficacy did appear to differ, with repositioning manoeuvres more likely to lead to resolution (78%) compared with Brandt-Daroff (53%) and Advice (42%) with an OR of 3.73 (95% CI 1.07 to 15.7; p=*0*.03). Significant differences were not noted in patient reported measures exploring dizziness burden (DHI) or balance confidence (ABC scale) between treatment groups. However, there were significant pre–post differences in dizziness burden (DHI) and balance confidence (ABC scale) in patients with *resolved,* but not *unresolved* BPPV, suggesting that dizziness burden and balance confidence scores improved from baseline to follow-up in only those with resolved BPPV (online supplemental material 5). A similar trend was also noted in gait speed data, that is, that only those with resolved BPPV showed improvements in gait speed.^8^

**Table 4:** Quantitative and qualitative data regarding randomisation

Treatment group	Falls	Recovery measures	Patients’ views	Therapists’ views
*Manoeuvres* Baseline DHI score: 36 (46) Drop-outs: 2 Withdrawals: 0	4	BPPV resolution 78% Change in DHI score: −19 GOSE score: 5.3 (1.37) EQ-5D index score: 0.81 (0.1)	*KC0309:* individuals need different treatment. Safety nets facilitated comfort with randomisation. *SM0604:* not worried about randomisation. Knew would get treated at some point.	*KC1908:* feels ok to randomise as more patients are being diagnosed, followed up/treated. Safety nets give reassurance. *KC0708:* ethically fine to randomise patients as no definitive treatment.
*Brandt-Daroff* Baseline DHI score: 15 (2) Drop-outs: 0 Withdrawals: 0	4	BPPV resolution 42% Change in DHI score: +3 GOSE score: 6.42 (1.01) EQ-5D index score: 0.89 (0.1)	*SG2112:* was not concerning that would be allocated by chance to treatment. *SG1708:* some concern randomisation might impact overall recovery. Safety nets mitigated this concern.	*SG2502:* prior to study would not opt to give patients Brandt-Daroff—felt out of comfort zone. *KC0104:* patients do not have an issue with randomisation. Explanation usually effective to resolve issues.
*Advice* Baseline DHI score: 22 (3) Drop-outs: 0 Withdrawals: 2	3	BPPV resolution 53% Change in DHI score: −12 GOSE score: 5.64 (1.76) EQ-5D index score: 0.91 (0.1)	*SG1606:* withdrew-did not realise was not going to have active treatment. *KC2307:* random allocation not a worry as hampered by other injuries. Perhaps different if dizziness had been more severe.	*SG1102*: felt responsible for patients if they are very dizzy and fall at home. *KC0408:* randomisation is important, but advice feels uncomfortable.

Quantitative data includes Dizziness Handicap Inventory (DHI) scores presented as median (IQR), percentage of patients with BPPV resolution at 12 weeks, mean (SD) Glasgow Outcome Score Extended (GOSE) and overall health as measured by the mean EQ-5D (SD). Qualitative data pertains to summary excerpts from patients’ and therapists’ transcripts. Falls were equally experienced among canal typologies.

BPPV, benign paroxysmal positional vertigo; EQ-5D, EuroQol 5 dimension.

**Table 5:** Joint display of qualitative and quantitative data pertaining to adverse events

Patient (group)	BPPV type	Adverse event timing and medication provided	Experience of assessment and treatment
KC2207 (Advice)	Bilateral posterior canal	Post-treatment at 12 week follow-up	Unsure whether manoeuvres acutely would be tolerable—unwell at follow-up
SM0607 (Advice)	Bilateral posterior canal	Postassessment at 4 and 12 weeks follow-up Required medication	Diagnostic and treatment manoeuvres not pleasant but medication helped. Manoeuvres resolved dizziness in the end.
SM0608 (Manoeuvres)	Bilateral posterior canal	Post-treatment acutely	N/A
SG0706 (Manoeuvres)	Unilateral posterior canal	Post-treatment acutely Required medication	Manoeuvres were not pleasant—resulted in an extra night in hospital. Has not had further dizziness so was worthwhile.
KC1406 (Brandt-Daroff)	Mixed posterior and horizontal	Post-assessment acutely	N/A

Treatment manoeuvres delivered to patients in the advice group were provided at 12 week follow-up. Individual patients are represented by their unique alphanumeric code and their intervention group.

BPPV, benign paroxysmal positional vertigo; N/A, not applicable (did not complete interview).

#### Qualitative findings

Of the 15 healthcare professionals and 26 patients completing semistructured interviews (tables2  3), the main findings were that acute assessment and treatment of post-traumatic BPPV was safe, practicable and tolerable. Convergence or agreement was noted on the importance of acute diagnosis in facilitating patients’ knowledge, understanding and confidence. However, divergence between healthcare professionals and patients was evident in views regarding randomisation; healthcare professionals reported some concern regarding randomising symptomatic patients to the advice arm, whereas patients in the advice arm noted that it was more practical to wait for repositioning manoeuvres due to complex limb and spinal fractures. Healthcare professionals and patients recommended several changes to the trial, specifically relating to outcome measures and recruitment processes. Fuller results can be found elsewhere.^20^

### Overview of integrated qualitative and quantitative findings

#### Recruitment

**Figure 2:**
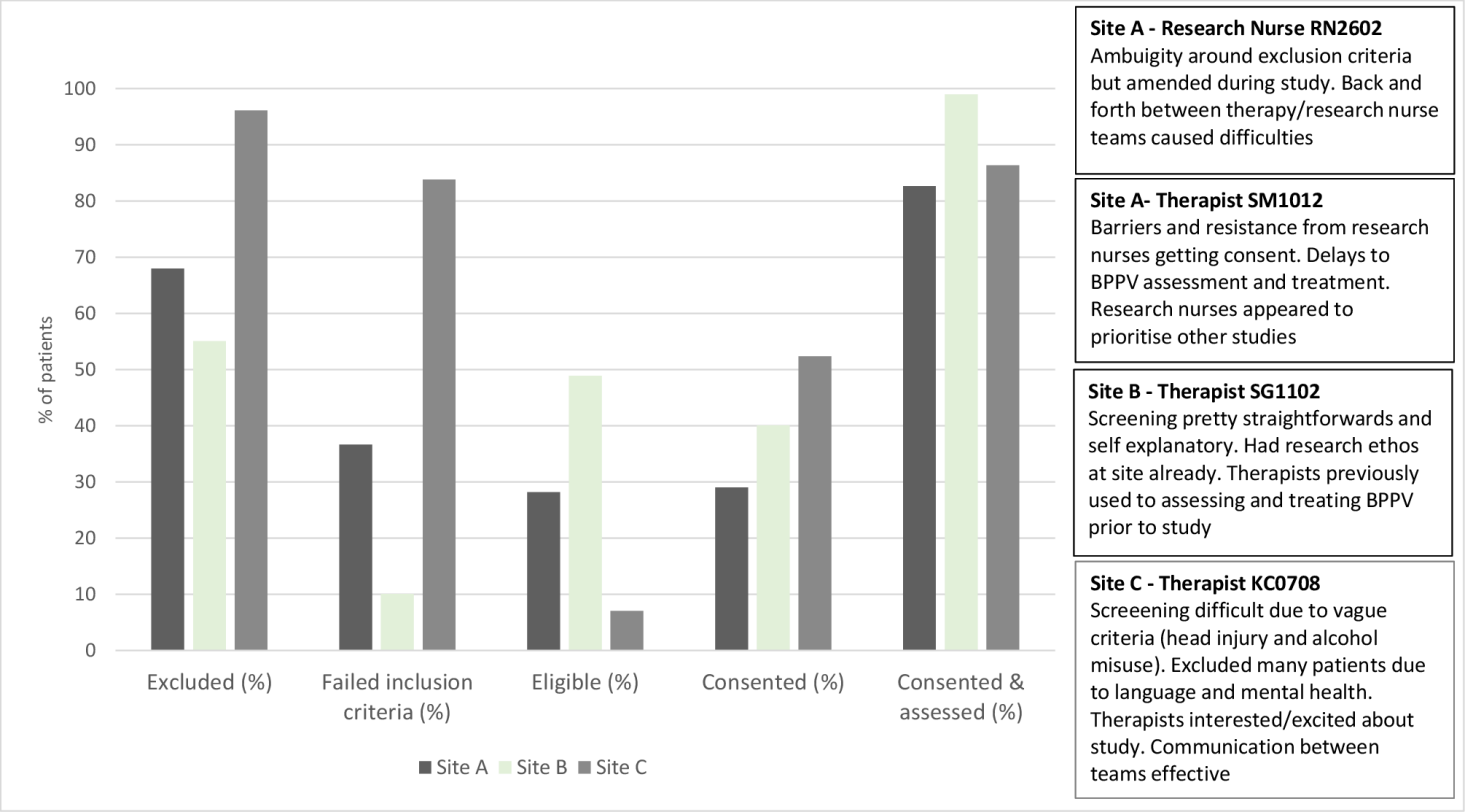
Joint display illustrating recruitment at the three sites during the feasibility trial. The bar chart represents the patients who were excluded, failed inclusion criteria, were eligible, consented and those who consented and were assessed across all three sites. Notes, which were derived from transcriptions of interviews with therapists and research nurses at specified sites, and which provide insight into their experiences of recruitment, are presented on the right, identified by participants' unique ID code. BPPV, benign paroxysmal positional vertigo.

Trial progression criteria set a priori stipulated that 30% of eligible patients were expected to consent, rising to 50% in the latter stages of the trial. Consent rates at sites varied between 29% and 52%. To further understand this variation and guide decision-making on trial progression, a joint display^41^ was developed using the quantitative and qualitative data (figure 2). These data, relating to screening patients for study inclusion, allow for similar conclusions to be drawn; that is ‘confirmation’ occurs between the data sets.^38 41^ The quantitative data suggest that sites A and C had higher rates of patient exclusion compared with site B, with greater proportions of patients failing to meet the inclusion criteria. Site C appeared to exclude a large proportion of patients due to a failure to meet inclusion criteria. The qualitative data appear to complement this; healthcare professionals at site C reported difficulties interpreting inclusion and exclusion criteria, particularly in relation to the terms ‘head injury’ and ‘alcohol misuse’. Interestingly, research nurses at site A also felt the screening criteria were ambiguous. However, this issue seemingly resolved during the study, possibly leading to a smaller proportion of patients being excluded at site A than at site C. The qualitative data gathered at site B indicate therapists perceived few problems with screening, reflected in the lower numbers of patients excluded.

The proportion of patients consented, and consented and assessed for BPPV was lowest at site A. The qualitative data offer some explanations; the process of consent and subsequent assessment caused communication and procedural difficulties between the research nurses and therapists. The highest consent rate was noted at site C, which was the last site to join the trial. The qualitative data indicate communication was effective at this site but do not offer further insights.

#### Randomisation

Exploring the acceptability of randomisation for patients and healthcare professionals was also a key aim of the feasibility study^8^ (table 4). Qualitative data suggest patients in the Brandt-Daroff and advice groups had some concerns regarding the impact of randomisation on their recovery, while therapists were apprehensive about randomising patients with a high symptom burden and an elevated falls risk to the advice group. Integration and interpretation of both data sets generated new insights. Contrary to therapists’ concerns, falls were equally frequent in all treatment groups, indicating falls were no more likely to occur in patients allocated to the advice group than in those who received repositioning manoeuvres. Additionally, patient-reported measures of subjective dizziness (DHI score), overall health outcome (EQ-5D index) and overall TBI recovery (Glasgow Outcome Score Extended (GOSE) score) were not significantly different between treatment groups (table 4 and online supplemental material 5), indicating patients were no more likely to have lower dizziness scores and health outcomes when treated with repositioning manoeuvres compared with advice. However, resolution of BPPV was markedly different in the manoeuvre group compared with Brandt-Daroff and advice groups (table 1), and data suggest that there were larger associated improvements in subjective and objective measures (table 4). A fully powered trial may be able to determine between-group differences in subjective dizziness scores, balance confidence, health outcomes and gait speed. Notably, the two patients who withdrew from the trial were both members of the advice group. They withdrew mainly due to the lack of ‘active’ intervention, which might suggest they and other patients were not accepting of randomisation procedures.

Thus, randomisation did not appear to *directly* impact changes in scores on patient-reported measures. However, since manoeuvres were linked to higher BPPV resolution and subsequent improvement in subjective and objective measures, an *indirect* link may exist between randomisation and markers of recovery.

#### Assessment and treatment of BPPV

To ascertain the possibility of progressing towards a fuller randomised controlled effectiveness trial, it was important to understand the safety of therapy-led assessment and treatment of BPPV via the incidence of AEs of vomiting. Table 5 shows those five patients who experienced AEs of vomiting and, where interviewed, their views on assessment and treatment. The quantitative data indicate that, of the five patients, four had either bilateral or mixed canal BPPV, although no patterns were noted regarding when the AEs occurred during the trial. The qualitative data suggest that despite the unpleasant nature of the procedures and the experience of vomiting (which in two cases required medication), patients remained positive about treatment. Notably, the patient in the advice group who experienced an AE during retesting at the end of the trial felt manoeuvres would have been somewhat overwhelming acutely.

**Figure 3:**
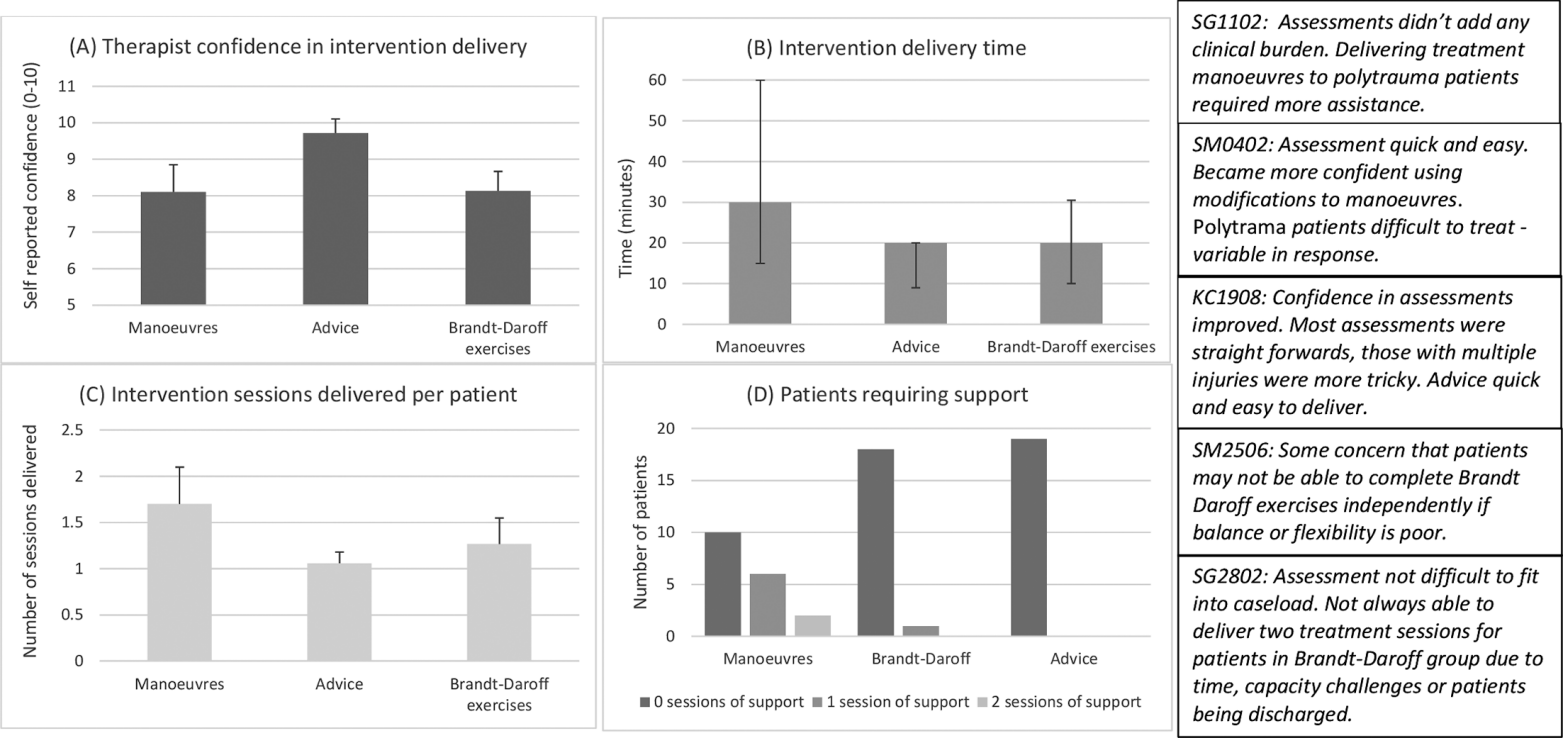
Joint display pertaining to treatment delivery in each intervention. Graph A refers to mean therapist confidence per group (as rated on a VAS scale from 0 to 10 with 10 most confident). Graph B refers to median time to deliver interventions per group. Graph C refers to the mean number of treatment sessions delivered per patient per group. Graph D refers to the number of patients per group requiring no sessions, one or two sessions with support from another therapist. Notes derived from transcripts of sections of therapists’ interviews pertaining to assessment and treatment are presented and are identified by unique alphanumeric codes. VAS, Visual Analogue Scale.

A further aspect of feasibility pertained to the acceptability of assessment and treatment procedures from the healthcare professionals’ perspectives, and whether such procedures could be implemented routinely. Quantitative and qualitative data were integrated in figure 3. Therapists at all sites reported that the assessments did not represent an additional clinical burden unless patients had additional injuries. In reference to delivering treatment, quantitative data imply the requirement to perform treatment manoeuvres with patients placed a greater burden on therapists. They required more time per treatment session with patients in the manoeuvre group (median treatment time of 30 min compared with 20 min per session with patients in the Brandt-Daroff and advice arms), additional staff support, plus more inpatient treatment sessions (figure 3). The qualitative data both support and refute this notion of greater treatment burden linked to repositioning manoeuvres. Therapists remarked that a proportion of patients allocated to the manoeuvre group had more complex injuries and, thus, were more difficult and time-consuming to treat, and had more variable treatment response. In reference to the number of treatment sessions delivered, the trial protocol stipulated that patients in the Brandt-Daroff and advice groups should have received two treatment sessions. Had this been delivered, all patients, irrespective of treatment group, would have had a similar number of treatment sessions. However, those in the Brandt-Daroff and advice groups received 1.2 and 1 sessions, respectively, which therapists attributed to limited time, capacity and imminence of discharge. Interestingly, these factors were not mentioned in reference to treating patients in the manoeuvre group, despite those patients receiving on average a higher number of sessions (1.7). Regarding the Brandt-Daroff group, the qualitative data suggest therapists had some concerns whether patients would be able to complete the exercises independently and be adherent to the programme at home. The quantitative data (online supplemental material 6) imply patients had varying levels of adherence, which was seemingly unrelated to resolution. Patients did not report difficulties completing the Brandt-Daroff exercises.

## Discussion

Assessment of intervention feasibility and acceptability is an integral part of complex intervention research.^23^ Experts in this field advocate the use of *mixed-methods* feasibility trials to inform progression to the next stage of intervention evaluation; however, it is more common than not for researchers to report quantitative and qualitative findings separately, rather than integrating the results.^40^ In this paper we have integrated qualitative and quantitative data from the first acute, prospective mixed-methods randomised feasibility trial investigating the management of BPPV in acute traumatic brain injury. Key to understanding whether the feasibility trial could progress to a more definitive trial was the search, within the integrated data, for in-depth answers to questions relating to recruitment, randomisation and the safety and practicability of BPPV assessment and treatment. Integration of data pertaining to recruitment provided some new insights into the appropriateness of trial progression. Seemingly, screening success varied between sites due to perceived ambiguity around inclusion and exclusion criteria. As noted by Keung *et al*,^43^ vague inclusion criteria can expose the study to bias.^43^ Spall *et al*^44^ recommended that explicit justification and careful wording of criteria should be provided in TBI trials, noting that terms such as ‘medical instability’ should be replaced with more specific objective criteria.^44^ Experts in the field of feasibility studies propose that strategies for maximising recruitment are often developed at the feasibility stage, and often from qualitative data yielded by early-stage trials.^45^ Thus, the wording of inclusion and exclusion criteria for a future, larger multicentre trial should be carefully considered to ensure efficiency and standardisation of recruitment processes. Involving patients, the public and relevant stakeholders would also be necessary to prevent any ambiguity in a future trial. Communication issues appeared to affect recruitment rates at one site. Communication around or relating to obtaining consent in clinical trials has received much attention;^46^,^48^ however, prior research has primarily focused on issues with interactions between patients and healthcare professionals. In this study, communication *between* healthcare professionals appeared to be a limiting factor in the consent process. At one site, and as encouraged by the study protocol, the research nurse team completed activities relating to consent and outcome measures, while the therapy team completed assessment and treatment activities. Since these different activities overlapped, effective communication and planning was crucial. This parsing of activities helped to ensure outcome assessors remained blinded; however, it presented a larger communication burden than expected. In a more definitive trial, considerations could be made to use dedicated assessors to avoid these communication problems, as has been used previously.^49^

Interestingly, consent rates were highest at site C, the last site to join the trial. Whether this reflects the motivation of staff in the early period of recruitment or that amendments made to the protocol earlier on in the study improved recruitment processes is unclear. Other evidence for variability in recruitment rates between the start and end of studies is not readily available. However, lower than expected *early* recruitment rates appear to be common.^50^ Thus, there may have been some individual factors influencing the favourable consent rate at site C. Such site-specific factors have been noted in other research.^50^,^52^ Common and relevant factors may include the availability of sufficient resources, the research infrastructure and recruiter characteristics. Indeed, the latter may well have been relevant in this study, as sites utilising therapists to consent patients (sites B and C) had higher rates of consent compared with site A, where research nurses consented patients. The qualitative data indicate therapists were interested and excited to participate in the study which may have translated into enthusiasm when discussing the study with patients. It is possible that this may have resulted in higher consent rates. Additionally, all therapists (regardless of whether they consented patients or assessed and treated patients) were trained in BPPV assessment and treatment prior to the study commencing and, thus, may have had a greater knowledge and confidence to draw on when explaining the cause, mechanisms and treatments of BPPV than the research nurses had. Future trials should consider these suppositions alongside other relevant research findings promoting strategies such as the use of trial managers, optimisation of information and education conveyed to investigators and development of trials grounded in existing clinical practices.^53 54^

The second key objective of this study was to develop a more comprehensive view of the acceptability of randomisation to different BPPV treatment arms. Integration of the study findings indicated that randomisation did not appear to directly influence secondary subjective and objective outcomes but was indirectly linked to these outcomes via BPPV resolution. Patients and healthcare professionals noted that the dizziness handicap inventory, the key subjective measure, was inappropriate for use in the acute trauma setting due to its focus on dizziness interference of community activities and participation. Additionally, some of the psychometric aspects of the DHI remain untested. Indeed, there are currently no robust tools designed to evaluate dizziness in acute settings.^55 56^ This feasibility study was also not powered to detect differences in outcomes between treatment groups. These potentially compounding factors may have contributed to the non-significant between-groups differences in subjective measure of treatment outcome. A future study with a larger sample size and a more robust outcome measure would be necessary to elucidate this and ensure best chances of identifying between-groups differences. Therefore, given (1) healthcare professionals’ concerns around randomisation, (2) the withdrawals from the advice group and (3) the potential indirect link between randomisation and scores on patient-reported outcomes, re-evaluation of the trial design is required ahead of a future, larger trial. Consideration needs to be given to the nature of the intervention arms. One approach may be to remove the advice arm or use an adaptive design whereby a priori adaptations, such as the removal of a treatment arm or changing the allocation of patients to a trial arm, can take place during the conduct of a trial dependent on ongoing data analysis.^57^ Regardless of the design of a future trial, involving therapists and patients in the planning stage would be important given the concerns raised in the present study around the inadequacy of advice as a treatment option.

Integration of data relating to the safety and acceptability of assessment and treatment procedures revealed that AEs were evenly dispersed throughout all treatment groups. This may prove useful in allaying some concerns regarding randomisation in a future trial. Notably most patients experiencing AEs had bilateral or mixed canal BPPV. The sample size of the current feasibility study precluded any further causal analyses; however, larger trials may be able to investigate this further. Evidence relating to vomiting in other BPPV trials is sparse, although rates that have been reported elsewhere are comparable.^58^ Interestingly, therapists did not indicate that the rate of AEs affected their views on the acceptability of diagnostic or treatment procedures. It would be useful to explore this more specifically within a larger trial, as previous research involving the management of BPPV by Emergency Physicians found that negative pre-trial experiences, such as worsening of symptoms or vomiting after sporadic BPPV interventions, were significant barriers to routine implementation of those interventions.^59^ To gain evidence about the clinical burden of implementing BPPV treatments, data on intervention monitoring were collected and integrated. Administering manoeuvres necessitated both more treatment sessions and more time per session. However, therapists felt less able to complete the required number of sessions (as per protocol) in the Brandt-Daroff and Advice arms. Whether this reflects therapists’ views of differing efficacy of the three interventions employed in the present study is unclear. Although there was no reported loss of equipoise in the study, as noted previously, concerns were voiced regarding randomisation of patients to the advice arm. The complexity of patients’ presentations, specifically the coexistence of limb and/or spinal fractures alongside TBI, also appeared to determine treatment time and staffing requirements. There is a paucity of literature on the resources required for treating BPPV in acute TBI patients. However, there are case reports about BPPV management in intensive care settings. Modified manoeuvres with assistance from the multidisciplinary team were employed in these studies.^60^ To support therapists delivering treatment for post-traumatic BPPV in future trials, as well as in routine practice, training a larger range of therapists and assistant therapists would be necessary to provide support for patients with complex or additional injuries. Patients allocated to the Brandt-Daroff group seemingly were able to complete the exercises at home independently, although with variable levels of adherence. It should be noted however that interviews did not include those with the lowest adherence or those over the age of 70 years, potentially limiting the generalisability of these interpretations. It should also be noted that the sampling strategy used may have introduced some selection bias. Data on how *accurately* patients performed the exercise at home were not collected but such data collection could be a focus for a future trial, alongside investigation of potential predictors of adherence to treatment. The data regarding adherence to Brandt-Daroff exercises in idiopathic BPPV are relatively sparse, although other authors report exercises are well tolerated according to completion of diaries.^61 62^

## Conclusions

Integration of qualitative and quantitative data from the feasibility trial has provided a richer and more comprehensive view of the possibility of progressing towards a definitive trial. Integration has also elucidated aspects of the trial requiring changes: (a) trial recruitment, (b) randomisation and trial design and (c) BPPV assessment and treatment. Respective strategies to consider for a future trial would include: (a) clarification of inclusion criteria and further understanding of specific resources available at individual sites, (b) consideration of alternative or adaptive trial designs to mitigate withdrawal of trial participants and/or researchers’ concerns about randomisation and (c) exploration of the mode and frequency of therapist training as well as modifications to BPPV management strategies. Such strategies would not only be useful in a future trial, but also ahead of the adoption of BPPV assessment and treatment in routine practice. Future research could also usefully evaluate any economic impact of early BPPV treatment.

## Supplementary material

10.1136/bmjopen-2026-117657 online supplemental file 1

## Data Availability

Data are available upon reasonable request.
