# Supplementary material for: Data from a UK-based multicentre randomised feasibility study investigating the management of post-traumatic benign paroxysmal positional vertigo: mixed-methods analyses
Source: BMJ Open. 2026 Jun 16;16(6):e117657. doi: 10.1136/bmjopen-2026-117657 (PMC13288678; doi:10.1136/bmjopen-2026-117657)
Supplement: online supplemental file 1 [file bmjopen-16-6-s001.docx]

**Supplementary material**

**Supplementary material 1 – Topic guide for patients participating in interviews**

**Participant Interview questions**

**Title: A qualitative study exploring the experience of taking part in a feasibility study investigating different treatments for BPPV in acute TBI.**

Chief Investigator: Rebecca Smith, Imperial College, Division of Brain Sciences, Charing Cross Hospital Campus, W6 8RF. Tel: 02033117042

**Introduction**

Thank you for agreeing to participate in this interview. The aim of the study is to explore your experience of participating in the dizziness in TBI feasibility trial. Your answers will be anonymous and will remain confidential. The interview will be audio recorded and I may take some notes whilst you are talking. Please keep in mind that you do not have to answer any questions you do not feel comfortable with, and we can stop the interview at any time.

**Motivation and Goals/Memory and Decision Making**

1. Why did you decide to take part in the trial? (i.e what was your motivation? please discuss any specific factors?)

**Knowledge**

1. How did you find the process of being asked to take part in research after your head injury? (i.e. how were you approached and consented?)
2. What did you think about the way in which we communicated with you? (how well did we explain the study?)
3. How well did you understand the trial from the information you received? (i.e. was there enough, was it easy to understand?)

**Emotion**

1. How did you feel about being randomly allocated to a treatment group? (e.g. Did it worry you? Did you feel it had any impact on your treatment/ recovery?)

**Environmental context and resources**

1. How do / did you feel about your dizziness being diagnosed in the hospital setting? (i.e was this timely? would this have been better done in the community? How did it fit in with the other care you received in hospital?)
2. What changes would you make to how your dizziness was diagnosed and treated which might help patients in a future trial?
3. What are your views on the treatments provided in the trial? (i.e were any sessions uncomfortable/ made your symptoms worse; did you decline to take part in any of the interventions?)
4. How capable did you feel about doing the treatments? (i.e. did the patients believe they could comply with what was being asked of them
5. If there were problems, were communication channels clear and easy to access?

**Skills / Beliefs about capabilities (of the clinicians)**

1. What was your experience of the staff delivering the treatments? (i.e. were the therapists the right staff to deliver the treatments? Were they perceived to be adequately trained?)

**Behavioural regulation**

1. If Brandt-Daroff exercise group – how did you keep up with the exercises prescribed to you by the ward team? (would you have any tips for future patients?)

**Beliefs about consequences**

1. Were there any benefits to being diagnosed and treated in the hospital? (i.e any benefits during the hospital stay and following discharge – were those tangible i.e. falls, going back to daily life, work etc)
2. Were there any negative consequences of being diagnosed and treated in the hospital?

**Trial design**

1. What was your experience of attending the follow up appointments? (i.e did you miss any? If so, why? Were they conveniently located?)
2. What was your experience of the questionnaires and tests you were asked to do? (i.e were they relevant to you; were they burdensome / too long / difficult to understand or complete?)
3. If we were to run this trial on a larger scale; what sort of things would you tell us to change?
4. Do you think this sort of research could run in other hospitals or other wards?

**Social influences**

1. How did your family/carer/friends view you being part of this research study?

Lastly, is there any further you would like to add which we have not covered?

**Thank you for participating in this interview.**

**Supplementary material 2 – Topic guide for healthcare professionals participating in interviews**

**NHS Healthcare Professional Interview questions**

**Title: A qualitative study exploring the experience of taking part in a feasibility study investigating different treatments for BPPV in acute TBI.**

Chief Investigator: Rebecca Smith, Imperial College, Division of Brain Sciences, Charing Cross Hospital Campus, W6 8RF. Tel: 02033117042

**Introduction**

Thank you for agreeing to participate in this interview. The aim of the interview is to explore your experiences of being involved in the feasibility trial. Your answers will be anonymous and will remain confidential. The interview will be audio recorded and I may take some notes whilst you are talking. Please keep in mind that you do not have to answer any questions you do not feel comfortable with, and we can stop the interview at any time.

**Opener question - ALL**

What was your general experience of taking part in the trial?

**Background questions - ALL**

1. Could you tell me a bit about your role was in the feasibility trial? (i.e. screening and consenting patients, assessment and treatment, outcome measures)

**Social professional role and identity – THERAPIST ONLY**

1. How did taking part in this study fit into your role as a therapist? (i.e. did you feel as a therapist you were the right person to do this?)

**Beliefs about capabilities – THERAPIST ONLY**

1. How did the assessment and treatment components of the trial fit within your current workload? (i.e. in terms of time? was this acceptable? Do you think this would be feasible to continue with in a longer trial?)
2. How would you rate your ability to diagnose and treat BPPV? (i.e. did you feel you had sufficient training?)

**Skills & Knowledge - Question 5, 8 & 9 THERAPIST ONLY, Question 6 & 7 ALL**

1. How did you find the completing the assessments? (were there any particular patients it was difficult to assess? Do the procedures need to be refined or adapted to make it more acceptable or relevant for future use?)
2. What are your views on the three different treatments we used in the trial? (i.e. did you understand why we used three different treatments? Were you comfortable with this?)
3. What are your views on patients being randomised to one of three different treatments? (what do you think patients felt about being randomised? We have ‘get out’ clauses in the protocol – i.e. patients being able to withdraw and an option for patients to see a Consultant specialising in dizziness if they need to when the study finishes – did these clauses make you feel any differently about randomising patients to the Brandt Daroff or Advice groups?)
4. What was your experience of delivering the treatments as per the protocol? (i.e could you always complete all the treatments and re-assessments? If not why not? did patients decline to complete treatments?
5. If you treated patients in the Brandt Daroff or advice group, were you always able to deliver two treatment sessions? (If not, why not?)
6. Similarly, with patients in the Brandt Daroff or Advice group, were you always able to re-assess patients after treatment? (If not, why not?)
7. What do you think the participants felt about the interventions? (i.e could they understand what you were asking them to do? Could they tolerate the interventions?)

**Beliefs about consequences - THERAPIST ONLY**

1. What were the consequences of the therapy team diagnosing and treating these patients during their hospital stay? (were there any benefits to the patients? To the team?)
2. How do you feel about treating these patients in the acute stage of their injury? (i.e was the acute setting an acceptable time/place to treat BPPV patients or would it be better to wait until they are out of hospital?)

**Environmental context and resources - ALL**

1. Were there clear communication channels if you were unsure of how to do something? (i.e was there adequate support if you needed it?)
2. This study is being run at different sites, have there been any specific barriers or facilitators that made it easy or difficult to participate in the trial? (i.e local or national guidelines about managing dizziness following head injury? would it be useful to have one therapist or nurse whose role just involved completing the non-clinical research activities?)

**Social influences - THERAPIST ONLY**

1. How did your colleagues view you taking on the role of diagnosing and treating BPPV? (other therapists/doctors/nurses/ward managers)

**Emotion - THERAPIST ONLY**

1. Can you describe any situations in which you were worried about managing BPPV in this population? (i.e medically/professionally/emotionally? Was there anything that made you feel uncomfortable?)

**Behavioral regulation - THERAPIST ONLY**

1. Outside of the trial are there any factors that would encourage you to continue with screening for BPPV? (local or national guidelines? Role models?)
2. Is there any further training that you or your team may require? (prompt: any procedures/guidelines/ways of working)

**Trial design questions - ALL**

1. What are your views on the overall design of the trial? (i.e. how patients were selected, the treatments that were used, the number and timing of the follow ups etc)
2. How did you find recruiting patients onto the trial? (i.e were there any specific barriers to recruiting, if so what were they? Did patients have sufficient cognitive skills to consent? Did we exclude too many patients? Did you have to exclude patients for a particular reason?)
3. What are your views on the outcome measures that the patients were asked to do? (i.e were they relevant? Were you able to complete the outcome measures in a timely manner? Do you think we change remove some? If so which ones?)
4. What are your views on this study being run as a full randomised controlled trial? (i.e could this be run in your setting again? Could it be run in other hospitals for a longer period? Could it run on other wards i.e. with head injury patients in A&E areas?)
5. What are your suggestions on how to improve the study? (Can you give practical examples of what we should do to improve it? i.e. change the paperwork, change REDcap?)

Lastly, is there any further you would like to add which we have not covered?

**Thank you for participating in this interview.**

**Supplementary material 3 – Flow Diagram**

Assessed for eligibility (n=2014)

Allocated to PRM (n=20)

Analysed (n=18)

Lost to follow up (n=0)

Discontinued intervention (treatment not working) (n=2)

Lost to follow up (n=0)

Discontinued intervention (n=0)

Allocated to Advice (n=19)

Allocated to Brandt-Daroff (n=19)

## Enrollment

## Allocation

## Follow-Up

Lost to follow up (not contactable) (n=2)

Discontinued intervention (n=0)

Excluded (n = 1818)

- Failure to meet inclusion criteria (n = 884)
- Declined to participate (n=144)
- Other exclusion criteria (n=790)

Assessed for BPPV (n=180)

Tested positive for BPPV and randomised (n=58)

Analysed (n=17)

ss

Analysed (n=19)

**Supplementary material 4 – Recruitment figures per site**

| **Site** | **Excluded** | **Failed inclusion criteria** | **Eligible** | **Consented** | **Consented & assessed** |
| --- | --- | --- | --- | --- | --- |
| Site A | 623/916 (68%) | 336/916 (37%) | 258/916 (28%) | 75/258 (29%) | 62/75 (83%) |
| Site B | 278/505 (55%) | 51/505 (10%) | 247/505 (48%) | 99/247 (40%) | 98/99 (99%) |
| Site C | 570/593 (96%) | 497/593 (84%) | 42/593 (7%) | 22/42 (52%) | 19/22 (86%) |

**Supplementary material 5 – Baseline and Follow up data and Inferential tests on patient reported measures**

Baseline and follow up data

| **Outcome** | **Baseline** | **4-week follow up** | **12 week follow up** |
| --- | --- | --- | --- |
| Dizziness Handicap Inventory (Median, IQR)  Manoeuvres  Brandt-Daroff exercises  Advice | 36 (26-72)  15 (7.5-44.5)  22 (16-56) | 36 (26-57.5)  18 (10-40)  29 (11.5-45.5) | 17 (3-43.5)  18 (9-31)  10 (0-32) |
| EQ-5D VAS (Mean, SD)  Manoeuvres  Brandt-Daroff exercises  Advice | 49.6 (26.1)  65.4 (15.7)  52.4 (22.9) | 48.9 (29.1)  73.5 (16.9)  67.5 (20.9) | 61.2 (20)  74.2 (17.6)  74.4 (19.5) |
| Glasgow Coma Score Extended  Manoeuvres  Brandt-Daroff exercises  Advice | NA  NA  NA | 5.1 (1.6)  5.4 (1.6)  4.9 (1.6) | 5.3 (1.4)  6.4 (1.0)  5.6 (1.8) |

Dizziness handicap inventory

A Kruskal-Wallis test was used to compare whether treatment group (Manoeuvres, Brandt-Daroff or Advice) had an effect on DHI scores. No effect was observed.

| **Timepoint** | **Chi Squared** | **Df** | **p-value** |
| --- | --- | --- | --- |
| Baseline | 5.0222 | 2 | 0.08 |
| T1 (4 weeks) | 1.3069 | 2 | 0.52 |
| T2 (12 weeks) | 1.5447 | 2 | 0.46 |

EQ-5D

A two way mixed ANOVA was used to determine whether treatment group (Manoeuvres, Brandt-Daroff or Advice) had an effect on EQ-5D scores. There was no interaction effect.

| **Effect** | **Df** | **Sum of Squares** | **Mean Square** | **F** | **P value** |
| --- | --- | --- | --- | --- | --- |
| Rx.group | 2 | 4177 | 2088.3 | 3.561 | 0.03 |
| Time | 1 | 4536 | 4536 | 18.067 | <0.001 |
| Rx.group*Time | 2 | 165 | 83 | 0.329 | 0.72 |

Glasgow Coma Score Extended

A Kruskal-Wallis test was used to test whether treatment group (Manovures, Brandt-Daroff or Advice) had an effect on GOSE scores. There was no effect observed.

| **Timepoint** | **Chi Squared** | **Df** | **P-value** |
| --- | --- | --- | --- |
| T1 (4 weeks) | 0.75573 | 2 | 0.68 |
| T2 (12 weeks) | 5.2935 | 2 | 0.07 |

**Supplementary material 6– Adherence to Brandt-Daroff exercises and views of patients completing them**

| **Participant** | **Age (Gender)** | **Confidence** | **Adherence** | **Resolved BPPV** | **Patient reported experience of exercises** |
| --- | --- | --- | --- | --- | --- |
| SM0903 | 36 (M) | 9 | 26/28 | No | Quick/easy to do independently. Dizziness reduced, stopped, then returned. |
| SG1708 | 65 (M) | 7 | 28/28 | Yes | Dizziness resolved after 10 days. Easy to do. Good to have something to do in hospital. |
| SM2610 | 81 (F) | 7 | 24/28 | No | N/A |
| SG1111 | 38 (M) | 8 | 27/28 | No | Exercise was straightforward. Unclear about how long was supposed to do exercise for. |
| SG0407 | 67 (M) | 5 | 28/28 | No | N/A |
| SM0807 | 61 (F) | N/A | 28/28 | No | N/A |
| SG0806 | 67 (M) | 2 | 28/28 | No | Exercises were new and unpleasant. Would have been helpful to go through exercises more in hospital. Felt they were helpful. |
| SG0508 | 20 (M) | 7 | 28/28 | No | N/A |
| SG1207 | 81 (M) | 4 | 7/28 | No | N/A |
| KC2608 | 28 (M) | 8 | 5/28 | Yes | N/A |

Abbreviations: M: Male; F: Female; Confidence was scored on a VAS from 0-10. Adherence to sessions was recorded by patients in a diary and was scored out of total 28 sessions.
