# Supplementary material for: Data from a UK-based multicentre randomised feasibility study investigating the management of post-traumatic benign paroxysmal positional vertigo: mixed-methods analyses
Source: BMJ Open. 2026 Jun 16;16(6):e117657. doi: 10.1136/bmjopen-2026-117657 (PMC13288678; doi:10.1136/bmjopen-2026-117657)
Supplement: Supplementary file 2 [file bmjopen-2026-117657.reviewer_comments.pdf]

PEER REVIEW HISTORY

BMJ Open publishes all reviews undertaken for accepted manuscripts. Reviewers are asked to complete a checklist review form and are provided with free text boxes to elaborate on their assessment. These free text comments are reproduced below.

ARTICLE DETAILS

Title (Provisional)

Data from a UK based multi-centre randomised feasibility study investigating the treatment of benign paroxysmal positional vertigo in acute traumatic brain injury: mixed methods analyses

Authors

Smith, Rebecca M; Sahu, Bithi; Burgess, Caroline; Beattie, Jenna; Newdick, Abby; Tahtis, Vassilios; Corcoran, Jeremy; Marsden, Jonathan; Seemungal, Barry

---

VERSION 1 - REVIEW

**Reviewer**	**1**
**Name**	**Harrell, Regan**
**Affiliation**	**University of Pittsburgh, Physical Therapy**
**Date**	**03-Apr-2026**
**COI**	**None**

Overall this study is well done and contributes to the field of rehabilitation for those who have experienced a traumatic brain injury. A few comments:

Intro:

Line 116: The way the sentence is phrased makes it seem like the Dix-Hallpike is the only provocative test. Please update for completeness.

Line 123: Please provide a citation and rationale for why Brandt-Daroff exercises are included for BPPV management especially when the AAOHNS BPPV CPG indicate that canilith repositioning maneuvers are up to 10x more effective than Brand-Daroff exercises.

Line 124: Please expand on what is meant by “advice”. In the CPG, the recommendation is for patient education regarding the impact of BPPV on their safety, potential for reoccurrence, etc. Is that what is included in the “advice” mentioned in this study

Methods:

Overall comment on methods: Were there any considerations for cognitive impairments during this study, especially given that some of the outcome measures, like the DHI and ABC require a strong understanding of a) the symptom (s) the person is feeling b) relating that to their own life. Given the high rates of cognitive impairments in this population, how did you know that the answers you were getting were accurate on those items and in the focus groups? Were all patients able to give their own consent? Or were proxies needed for some. Could there be different results in patients that cognitively can't provided their own consent which may indicate a more severe brain injury?

Line 156: Were clinicians allowed to select what repositioning maneuvers to use or per protocol could they only use the ones listed?

Lines 173/174: Unclear what the sentence "The trial... was not optimal" is attempting to convey. Please update for clarity

In Table 1, the Functional Ambulation Category is mentioned as a baseline characteristic. Please describe this in the text, and comment on the number of subjects who were independent with ambulation given the severity of their brain injury and if that might be affecting the results at all

Line 241: Can you provide clarity as what the research team was providing consensus on? Was it on the coding of the transcripts or the Framework? The current sentence is unclear.

Results:

Table 4: It would be interesting to know the canal typography of those with the fall in the group and those who had BPPV resolve. Horizontal canal BPPV has the highest rate of spontaneous resolution- does the data in this study support that? For those that experienced a fall, what other impairments did they have? In Table 1 for the baseline characteristics the only functional measure provided is the Functional Ambulation Category. Where the modified DGI scores similar across groups? This could have also affected the fall rates

Line 319: Unclear what the sentence "This trend... speed data" is conveying. That dizziness can affect gait speed or that BPPV resolution does?

Line 328: Fascinating about the advice arm, since advice is not described in the paper it is confusing to know if a patient was being counseled on the increased fall risk related to BPPV especially in the setting of other factors that can contribute to falls (weakness, non -weight bearing, etc) and the patients were able to make informed decisions about remaining in the advice arm. It would also be nice, as mentioned earlier, to know their cognitive status when making these statements

Line 379-382: See comments regarding Table 4

Line 406: Does adverse effect only refer to vomiting or are there other adverse events? It is unclear from the text

Line 413: Did the patient who experienced an AE at the end of the trial, was this during a repositioning maneuver/retesting? It is unclear when the AE occurred

Discussion:

Line 526/527: Given the relatively small sample size and lack of clarity of how similar the patients were functionally before randomization, this statement feels a bit over reaching.

Line 578: “The complexity of patient’s presentations...staffing requirements” is a really interesting statement, there doesn’t appear to be data in this paper to support that statement. Please provide a reference or indicate where in the data this was shown

References:

Reference 20 is incomplete

**Reviewer**	**2**
**Name**	**Bruintjes, Tjasse**
**Affiliation**	**Gelre Hospital Apeldoorn, Apeldoorn Dizziness Centre**
**Date**	**11-Apr-2026**
**COI**	**None**

This paper presents data from of a feasability study investigating the treatment of post-traumatic BPPV. It focuses on trial recruitment, randomisation, assessment and treatment. Interestingly an extensive study protocol has been published in 2020, whilst the study results have been published in 2024. The 2024 paper concludes that there is strong evidence for the feasability of a future trial.

The conclusion of the current paper is that the trial data provide 'richer and more comprehensive view of the possibility of progressing towards a definitive trial' (which sounds rather vague to me). Luckily, some specific (and useful) changes that are required for such a trial are listed.

This is acutally a nice study, but it seems to me the main formation in this paper has already been presented in the two previous studies. A lot of information is therefore repeated— is it really necessary to make this article so extensive? I printed the article plus the supplement, and it resulted in 82 pages! It seems to me that it should be possible to convey the message in a more concise form.

Some minor points: 1. It seems unethical to me to include ' advice' as a treatment option (since CRM is such an effective treatment). The guideline states that observation is an option in some (not all) patients, but it strongly favors CRM as initial treatment.

2. The authors rightfully conclude that the DHI is inappropriate for use in the acute setting.

VERSION 1 - AUTHOR RESPONSE

Reviewers' comments	Our response	Page and line location
Line 116: The way the sentence is phrased makes it seem like the Dix-Hallpike is the only provocative test. Please update for completeness.	Thank you. We have re-phrased this. The text now reads:<br><br>"During a provocative test, Dix Hallpike or Supine head roll test"	Page 5, Line 124
Line 123: Please provide a citation and rationale for why Brandt-Daroff exercises are included for BPPV management especially when the AAOHNS BPPV CPG indicate that<br>canilith repositioning maneuvers are up to 10x more effective than Brand-Daroff exercises	Thank you for this comment. In our experience, Brandt Daroff exercises are still recommended by clinicians who do not have the skills, knowledge or confidence to perform manoeuvres – this was also shown in a survey study by Male et al. in 2018.<br><br>We acknowledge the sentence could be re-worded to reflect the differences in effectiveness so have edited the sentence to read:<br><br>"Repositioning manoeuvres are recommended for treatment of idiopathic BPPV [16], however Brandt-Daroff exercises and watchful waiting, with patient education, continue to be used as treatment [19], despite unfavourable evidence [16].	Page 5<br><br><br><br><br><br><br><br><br><br><br><br><br><br>Line 131
Line 124: Please expand on what is meant by "advice". In the CPG, the<br>recommendation is for patient education regarding the impact of BPPV on their safety, potential for reoccurrence, etc. Is that what is included in the "advice" mentioned in this study	Thank you – in the CPG, observation is noted as an option for the management of 'some' patients with BPPV – alongside patient education materials. We have edited the sentence to read:	Page 5

	"Repositioning manoeuvres are recommended for treatment of idiopathic BPPV [16], however Brandt-Daroff exercises and watchful waiting, with patient education, continue to be used as treatment [19], despite unfavourable evidence [16].<br><br>We have also clarified what we termed as advice in our study: … advice [verbal and written advice relating to sitting or standing up slowly, being adequately hydrated]).	Line 131<br><br>Page 7<br><br>Line 174
Overall comment on methods: Were there any considerations for cognitive impairments during this study, especially given that some of the outcome measures, like the DHI and ABC require a strong understanding of a) the symptom (s) the person is feeling b) relating that to their own life. Given the high rates of cognitive impairments in this population, how did you know that the answers you were getting were accurate on those items and in the focus groups? Were all patients able to give their own consent? Or were proxies needed for some. Could there be different results in patients that cognitively can't provided their own consent which may indicate a more severe brain injury?	Thank you for this interesting question. Patients who did not have capacity to consent were recruited via a consultee. 3/58 patients were recruited via this method.<br><br>We were also concerned about patients' ability to report their own symptoms/impairments – so we used the Awareness questionnaire (which is completed by the patients, and by either a family member of a clinician) to explore whether patients were accurately able to report symptoms or impairments.<br><br>On analysis of the data, we did not find significant differences between patients' awareness and that of their family members or clinicians indicating on the whole, patients had sufficient insight to report their impairments.<br><br>We also used the MoCA to screen for cognitive impairments – the whole sample baseline	N/A

	mean score was 27/30 indicating normal cognitive function.	
Line 156: Were clinicians allowed to select what repositioning maneuvers to use or per protocol could they only use the ones listed?	They were only trained and permitted to use the manoeuvres that are listed on Line 75.	N/A
Lines 173/174: Unclear what the sentence "The trial… was not optimal" is attempting to convey. Please update for clarity	Thank you – we have edited this sentence to read:<br><br>"The trial was designed and delivered following discussions with patients, who reported that acute care for patients with post-traumatic vestibular dysfunction was not optimal"	Page 8<br><br>Line 194
In Table 1, the Functional Ambulation Category is mentioned as a baseline characteristic. Please describe this in the text, and comment on the number of subjects who were independent with ambulation given the severity of their brain injury and if that might be affecting the results at all	Thank you. We have described this more fully in the text beneath Table 1. The text now reads:<br><br>"Abbreviations: GCS (at scene), Glasgow Coma Score; DHI, Dizziness handicap inventory; FAC, functional ambulation category (six point clinician rated scale ranging from non-functional to independent on all surfaces)…"<br><br>The numbers of patients who were independent are shown in the table, for each group.	Table 1.<br><br>Line 319
Line 241: Can you provide clarity as what the research team was providing consensus on? Was it on the coding of the transcripts or the Framework? The current sentence is unclear.	Thank you – we have amended this sentence to read:<br><br>"Framework drafts were discussed amongst the research team and refined until a consensus regarding the Framework was reached"	Page 11<br><br>Line 264
Table 4: It would be interesting to know the canal typography of those with the fall in the group and those who had BPPV resolve. Horizontal canal BPPV has the highest rate of	The canal typography for fallers was as follows; bilateral posterior canal BPPV: 5; unilateral posterior canal BPPV: 5 and Mixed horizontal and	Table 4

spontaneous resolution- does the data in this study support that? For those that experienced a fall, what other impairments did they have? In Table 1 for the baseline characteristics the only functional measure provided is the Functional Ambulation<br>Category. Where the modified DGI scores similar across groups? This could have also affected the fall rates	posterior canal BPPV: 1. We have added a line to Table 4 to this effect.<br><br>Regarding your question around resolution of horizontal canal BPPV, this is an interesting thought, however we did not have enough patients with horizontal canal BPPV to test whether they were more likely to resolve spontaneously compared to those with posterior canal BPPV.<br><br>The total modified DGI scores were not significantly different at baseline between canal typology (51.45±11.53 for unilateral BPPV and 46.28±17.87 for bilateral BPPV). A two way mixed ANOVA (Group [Manoeuvres vs Brandt-Daroff vs Advice] and Time [baseline vs 12 week follow up]) showed no significant interaction on dynamic balance as measured by the mDGI.	
Line 319: Unclear what the sentence "This trend… speed data" is conveying. That dizziness can affect gait speed or that BPPV resolution does?	We have clarified this:<br><br>"A similar trend was also noted in gait speed data, i.e. that only those with resolved BPPV showed improvements in gait speed"	Page 15<br><br>Line 347
Line 328: Fascinating about the advice arm, since advice is not described in the paper it is confusing to know if a patient was being counseled on the increased fall risk related to BPPV especially in the setting of other factors that can contribute to falls (weakness, non - weight bearing, etc) and the patients were able to make informed decisions about remaining in the advice arm. It would also be nice, as mentioned earlier, to know their cognitive status when making these statements	Thank you – we hope the earlier clarifications about what was included in the advice arm have helped.	Page 7<br><br>Line 174

Line 379-382: See comments regarding Table 4	We have added a sentence regarding the distribution of falls amongst canal typology.	Table 4
Line 406: Does adverse effect only refer to vomiting or are there other adverse events? It is unclear from the text	Yes - We have now clarified this:<br><br>"it was important to understand the safety of therapy-led assessment and treatment of BPPV via the incidence of adverse events (AE) of vomiting"	Page 21<br><br>Line 452
Line 413: Did the patient who experienced an AE at the end of the trial, was this during a repositioning maneuver/retesting? It is unclear when the AE occurred	This was at re-testing. We have amended the text to read:<br><br>"Notably, the patient in the advice group who experienced an AE during re-testing at the end of the trial felt manoeuvres would have been somewhat overwhelming acutely."	Page 21<br><br><br>Line 460
Line 526/527: Given the relatively small sample size and lack of clarity of how similar the patients were functionally before randomization, this statement feels a bit over reaching.	We acknowledge this concern and have re-phrased the text to read:<br><br>"Integration of the study findings indicated that randomisation did not appear to directly influence secondary subjective and objective outcomes but was indirectly linked to these outcomes via BPPV resolution"	Page 25<br><br><br>Line 565
Line 578: "The complexity of patient's presentations…staffing requirements" is a really interesting statement, there doesn't appear to be data in this paper to support that statement. Please provide a reference or indicate where in the data this was shown	This was noted in qualitative data – as shown in Figure 3.	Figure 3
Reference 20 is incomplete	Thank you we have amended this.	Page 36
A lot of information is therefore repeated— is it really necessary to make this article so extensive? I printed the article plus the supplement, and it resulted in 82 pages! It seems to me that it should be possible to convey the message in a more concise form.	Thank you for this feedback. We are aware it is a lengthy article – we have therefore made efforts to reduce the word count, particularly in the introduction, methods and discussion sections.	As identified by the strikethrough function
Some minor points: 1. It seems unethical to me to include ' advice' as a treatment option	Thank you – Yes we do understand your concern.	

(since CRM is such an effective treatment). The guideline states that observation is an option in some (not all) patients, but it strongly favors CRM as initial treatment.	However, there are no data relating to treatment effectiveness in the TBI cohort – and interestingly as the study found, these patients were at high risk of recurrence following acute treatment. It therefore remains unknown whether it is more beneficial to wait, and treat in the sub-acute phase. There is fuller justification for the trial arms in the paper reporting the quantitative data from the trial. As the reviewer points out, the article is already saturated, and we feel adding this depth of detail would not be appropriate. We have signposted the reader to this in the paper:<br><br>"Further trial design and methodological details can be found in prior publications"	Page 7<br><br>Line 183
2. The authors rightfully conclude that the DHI is inappropriate for use in the acute setting.	Thank you – we are exploring the development of a new measure.	N/A
Please revise the first bullet point of the 'Strengths and limitations of this study' section of your manuscript (after the abstract). This section should contain up to five short bullet points, no longer than one sentence each, that relate specifically to the methods. The novelty, aims, results or expected impact of the study should not be summarised here.	Thank you – we have revised this to read<br><br>"This paper uses a mixed methods approach to report quantitative and qualitative trial data from a multi-centre feasibility study investigating assessment and treatment of BPPV in acute TBI."	Page 3<br><br>Line 88
Please replace the current CONSORT checklist with a copy of the CONSORT extension for pilot and feasibility trials checklist indicating the page/line numbers of your manuscript where the relevant information can be found (http://www.bmj.com/content/355/bmj.i5239)	We have included a populated CONSORT extension for pilot and feasibility studies as an attachment.	N/A
Please include the setting in the title.	We have re-phrased the title to read	Page 1

	"Data from a UK based multi-centre randomised feasibility study investigating the management of post-traumatic benign paroxysmal positional vertigo: mixed methods analyses"	

VERSION 2 - REVIEW

**Reviewer**	**1**
**Name**	**Harrell, Regan**
**Affiliation**	**University of Pittsburgh, Physical Therapy**
**Date**	**14-May-2026**
**COI**	

not applicable
